# Missing Link: Barr and Needham Respond

**Published:** 2005-10

**Authors:** Dana B. Barr, Larry L. Needham

**Affiliations:** National Center for Environmental Health Centers for Disease Control and Prevention, Atlanta, Georgia, E-mail: dbarr@cdc.gov

In January 2004, the Monsanto Company contacted our laboratory at the Centers for Disease Control and Prevention (CDC) regarding their concern about the association between alachlor (a Monsanto product) exposure and semen quality reported by Swan et al. (2003). As a result, we provided Monsanto with detailed information about our methodology for alachlor exposure assessment by measuring its urinary metabolite alachlor mercapturate (AM). In addition, we participated in a study in which Monsanto sent 25 urine samples to the CDC for analysis. Monsanto had spiked 15 of these samples with AM (< 1.5 ng/mL), and 10 unspiked samples were collected from a field study; all were blinded to the CDC. We did not detect AM (> 0.1 ng/mL) in any of the field samples, including one with an alleged interferent; thus, the method did not produce false-positive results. For the spiked urine samples, the CDC and Monsanto measurements showed excellent correlation (*r* = 0.9881; *p* < 0.0001), although the Monsanto measurements averaged about 30% higher. Similarly, the CDC sent samples representing a broader range of concentrations (~ 1–100 ng/mL) to Monsanto for blinded analysis; again, the results were comparable.

At Monsanto’s request, residual samples from those originally tested by Swan et al. (2003) were sent to them for analysis. Because of sample volume constraints, Monsanto pooled individual samples to produce three samples with concentrations of < 0.1 ng/mL, approximately 0.2 ng/mL, and approximately 3 ng/mL. Monsanto did not detect AM in any of the pooled samples; thus, they concluded that the CDC obtained false-positive results possibly caused by a putative interferent. We suggested that Monsanto use the CDC method in its laboratory to assess whether they observed the interferent. Although Monsanto originally agreed to do this, they reportedly did not do so.

The addition of confirmation ions does increase confidence in measurements, although the method used by Swan et al. (2003) was peer-reviewed, published in *Analytical Chemistry*, and included many components that produce highly reliable results (Olsson et al. 2004). We have since acquired technology that allowed us to measure AM with a similar limit of detection while including confirmation ions. Using both the older method (Olsson et al. 2004) and a newer one (Norrgran et al., in press), we analyzed 14 properly archived samples that were split from samples originally analyzed and reported by Swan et al. (2003) and compared all data. In these samples, the AM levels were similar to those previously obtained (*r* = 0.9912; *p* < 0.0001) (Norrgran et al., in press) and showed good agreement using either method (*r* = 0.9999; *p* < 0.00011) (Norrgran et al., in press). We recently shared with Monsanto chromatograms of a urine sample with low levels of AM as determined by all three analyses and provided sufficient information with which to evaluate the methodology. Furthermore, we offered to discuss these new results with Monsanto, but they have not accepted this offer.

Finding AM concentrations in urine samples collected in 2000 from men in Missouri is not unlikely. Several studies have detected alachlor with high frequency in Midwestern groundwaters and surface waters (Battaglin et al. 2000; Lerch and Blanchard 2003) near the time and location our sampling occurred. Thus, although we do not frequently detect AM in general population samples, we were not surprised to find it in urine samples collected from this region. Also, contrary to Gustafson’s claim, we have not yet analyzed any field samples from other agricultural areas using our new method.

We strive to present quality human exposure assessment data. We have been assessing alachlor-related exposures since 1994; in fact, we were the first to report that AM was the primary human metabolite of alachlor (Driskell et al. 1996). Our laboratory uses both the highest caliber instrumentation and isotopically labeled internal standards, which result in high-quality, validated exposure-assessment methods capable of producing reliable and consistent results. Furthermore, our laboratory is certified to analyze human biological samples according to the Clinical Laboratory Improvement Amendment (1988), which requires extensive quality control and assurance, semiannual blinded proficiency testing, continued verification and documentation of operational parameters, and recertification every 2 years.

We do not know why Monsanto did not obtain similar results when analyzing pooled urine samples left over from the original analyses. Possible false-negative analyses could result from multiple confirmation ions that limit the sensitivity of detecting low concentrations, degradation of AM in the samples that had undergone several thaw-refreeze cycles, or inadvertent dilution of AM during the pooling process. However, the results from our analysis of properly archived specimens from 14 of the same persons from the original study provide strong evidence that our first analyses were, indeed, correct. Perhaps, when we have more details on Monsanto’s methodology and sample handling procedures, we can further explore potential reasons for the discrepancy between our results.
